# Unraveling the compromised biomechanical performance of type 2 diabetes- and Roux-en-Y gastric bypass bone by linking mechanical-structural and physico-chemical properties

**DOI:** 10.1038/s41598-018-24229-x

**Published:** 2018-04-12

**Authors:** Carlos Marin, Georgios Papantonakis, Kathleen Sels, G. Harry van Lenthe, Guillaume Falgayrac, Roman Vangoitsenhoven, Bart Van der Schueren, Guillaume Penel, Frank Luyten, Katleen Vandamme, Greet Kerckhofs

**Affiliations:** 1 Skeletal Biology and Engineering Research Center, Department of Development and Regeneration, KU Leuven, Leuven, Belgium; 2 Prometheus - Division of Skeletal Tissue Engineering Leuven, KU Leuven, Leuven, Belgium; 3 Biomaterials – BIOMAT, Department of Oral Health Sciences, KU Leuven, Leuven, Belgium; 4 Biomechanics Section, Department of Mechanical Engineering, KU Leuven, Leuven, Belgium; 5 Lille University, Littoral Côte d’Opale University, EA 4490, PMOI, Physiopathologie des Maladies Osseuses Inflammatoires, F-59000 Lille, France; 6 Clinical and Experimental Endocrinology, Department of Clinical and Experimental Medicine, KU Leuven, Leuven, Belgium; 7 Biomechanics lab, Institute of Mechanics, Materials, and Civil Engineering, UCLouvain, Louvain-la-Neuve, Belgium

## Abstract

Type 2 diabetes mellitus (T2DM) is a metabolic disorder associated with obesity and hyperglycemia. Roux-en-Y gastric bypass (RYGB) surgery is a common treatment for severely obese patients and T2DM. Both RYGB and T2DM are linked to increased skeletal fragility, though the exact mechanisms are poorly understood. Our aim was to characterize the structural, mechanical and compositional properties of bones from diet-induced obese and RYGB-treated obese (bypass) mice to elucidate which the exact factors are contributing to the increased skeletal fragility. To achieve this, a combinatory approach including microfocus X-ray computed tomography, 3-point bending, finite element modeling and Raman spectroscopy, was used. Compared to aged-matched lean controls, the obese mice displayed decreased cortical thickness, trabecular bone loss, decreased stiffness and increased Young’s modulus. For the bypass mice, these alterations were even more pronounced, and additionally they showed low mineral-to-matrix ratio in the cortical endosteal area. Accumulation of the advanced glycation end-product (AGE) pentosidine was found in the cortex of obese and bypass groups and this accumulation was correlated with an increased Young’s modulus. In conclusion, we found that the increased fracture risk in T2DM- and post-RYGB bones is mainly driven by accumulation of AGEs and macro-structural alterations, generating biomechanical dysfunctionality.

## Introduction

Obesity is a complex disorder that has become an epidemic of great impact in our society. It is the result of imbalanced food intake and energy expenditure^1^. Type 2 diabetes mellitus (T2DM) is one of the most common comorbidities resulting from obesity, and is characterized by hyperglycemia, through insulin resistance and islet β-cell dysfunction^2^. T2DM is also known to affect bone homeostasis and quality, to decrease bone strength^3^ and to increase fracture risk^4^. Gastric bypass surgery is a valuable approach for the treatment of patients suffering from T2DM and obesity, resulting in reversal of the hyperglycemia, and rapid and significant weight loss^5^. Currently, the Roux-en-Y gastric bypass (RYGB) procedure has been associated with the most effective drop in bodyweight and a higher remission of diabetes, compared to other procedures such as laparoscopic gastric banding and lifestyle intervention^6,7^. In spite of these benefits, complications such as calcium malabsorption, bone loss, altered cortical and trabecular structure, decreased failure load and increased fracture risk have been reported^8–10^.

Clinical research has indicated that, despite a normal or high bone mineral density, fracture risk in T2DM patients is significantly higher at locations such as the hip and vertebrae, compared to non-diabetic subjects^11,12^. Skeletal fragility is determined by bone quality, which comprises material, mechanical and structural properties of the bone tissue^13^. An early study from Saito *et al*. (2006) demonstrated that bone quality is compromised in T2DM WBN/Kob rats through deterioration in the material properties of their bones. The authors showed the accumulation of pentosidine, an advanced glycation end-product (AGE) agent, in the femora of the rodents^14^. AGEs are generated by excessive non-enzymatic cross-linking of the collagen, known to negatively influence the material and mechanical properties of bone tissue through stiffening of the matrix and hindering processes such as bone mineralization^14–17^. Moreover, it was also highlighted in the study that after 3-point bending mechanical tests, the ultimate load, stiffness and work-to-failure of the T2DM femora were significantly decreased compared to non-diabetic controls, evidencing compromised mechanical properties and increased fracture risk under T2DM conditions^14^. Likewise, estimated bone strength in terms of ultimate load was found decreased in morbidly obese patients that underwent RYGB surgery^9^. It was suggested that the poor mechanical performance of the RYGB bone is a consequence of compromised structural properties post-surgery, specifically decreased cortical thickness, low trabecular number and increased trabecular separation^9^. Compromised bone structural properties, determined by means of microfocus computed tomography (microCT), have also been reported for C57BL/6 mice models of high-fat diet-induced T2DM^18,19^, and these properties have been previously associated with decreased bone mechanical properties^20,21^.

Furthermore, it has been previously demonstrated that the molecular composition also plays a role in the quality and biomechanical behavior of bone tissue^22^. In ageing Sprague-Dawley rats and baboons, the indentation modulus and hardness of femora were correlated with Raman microspectroscopy-based mineral-to-matrix and carbonate-to-phosphate ratios, contributing to the detriment of biomechanical behavior of bone^23,24^. These studies demonstrated that bone composition and intrinsic mechanical properties are interrelated and negatively influenced by ageing effects.

Despite the current knowledge on the impact of T2DM and RYGB surgery on bone tissue properties and fracture risk, the exact causes of these detrimental effects are still not well understood. Therefore, the main objective of this study was to characterize bone’s structural, mechanical and compositional properties and to decipher the exact factors causing the compromised bone quality and increased fracture risk under T2DM- and post-RYGB conditons. In order to achieve this, a combinatory approach by means of both experiments and computational modeling was adopted.

## Results

### Physiological characteristics altered by T2DM and RYGB

**Figure 1:**
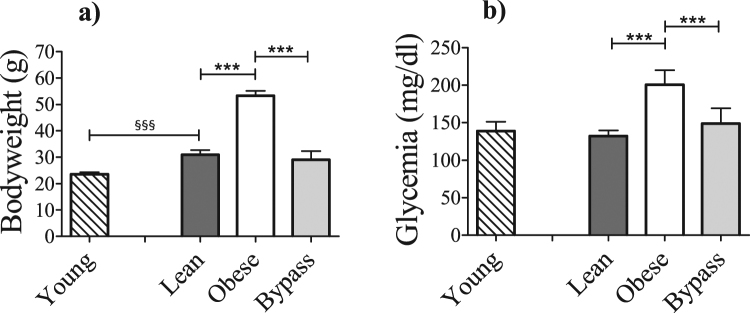
(**a**) Bodyweight and (**b**) fasting glycemia of young, lean, obese and bypass mice. ^§§§^p < 0.001 when comparing the young and age-matched lean group; ***p < 0.001 when comparing the obese, lean and bypass groups. n = 6/group.

After 22 weeks of high-fat diet treatment, the obese mice displayed a significant increase in bodyweight and fasting blood glucose compared to lean controls (Fig. 1), indicating obesity-driven T2DM conditions with poor glucose handling. The bypass mice showed similar bodyweight and glycemia values compared to lean control animals, though all significantly reduced compared to their obese counterparts. When young and lean mice were compared, the latter displayed significantly higher bodyweight and similar blood glucose levels.

### Cortical and trabecular bone architecture are compromised by T2DM and RYGB

**Figure 2:**
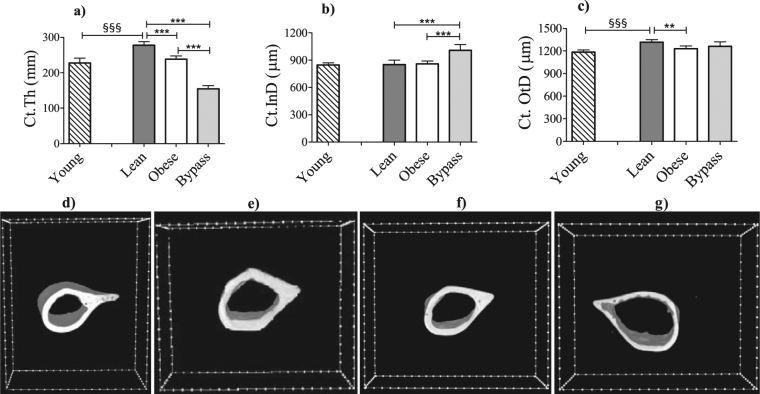
MicroCT-based quantification of the cortical bone structural parameters (upper panels): (**a**) Ct.Th, (**b**) Ct.InD and (**c**) Ct.OtD for young, lean, obese and bypass femora. 3D renderings of the cortex of (lower panels): (**d**) young, (**e**) lean, (**f**) obese and (**g**) bypass femora. ^§§§^p < 0.001 when comparing the young and age-matched lean group; **p < 0.01 and ***p < 0.001 when comparing the obese, lean and bypass groups. n = 6/group.

To investigate the role of the macro-structure in the skeletal fragility of our obese and bypass mice, the cortical and trabecular compartments of the femora were evaluated by means of microCT. Ct.Th was found to be significantly decreased in obese mice compared to lean mice and an even lower thickness was observed in bypass mice compared to both previous groups (Fig. 2). It is important to highlight that, relative to lean controls, the obese group showed a decreased Ct.OtD and the bypass group presented an increased Ct.InD, indicating impairment of the bone apposition process for both mice groups. Regarding the ageing effect on cortical bone, lean mice showed a significantly increased Ct.Th and increased Ct.OtD compared to young animals, though an unchanged Ct.InD.

**Figure 3:**
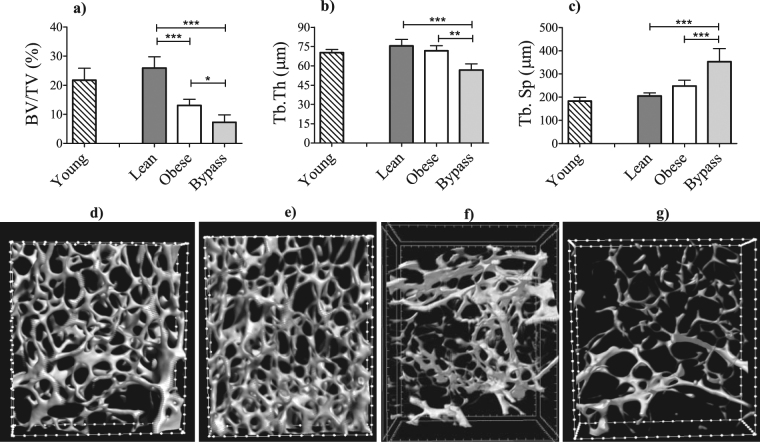
MicroCT-based quantification of the trabecular bone structural parameters (upper panels): (**a**) BV/TV, (**b**) Tb.Th and (**c**) Tb.Sp of young, lean, obese and bypass mice femora. 3D renderings of the trabecular area of (lower panels): (**d**) young, (**e**) lean, (**f**) obese and (**g**) bypass femora. **p < 0.01 and ***p < 0.001 when comparing the obese, lean and bypass groups. n = 6/group.

Relative to lean controls, the trabecular BV/TV was drastically reduced in obese mice and even more so in bypass mice (Fig. 3). Additionally, the BV/TV difference between obese and bypass mice reached statistical significance (p < 0.05). Furthermore, the trabeculae were the thinnest in bypass animals and presented significantly increased Tb.Sp compared to both obese and lean counterparts, all these suggesting potential bone remodeling alterations. Femora from young mice had the tendency to be slightly reduced in all trabecular parameters when compared to lean controls without reaching statistical significance.

### Bone biomechanical and material properties are affected by T2DM and RYGB surgery

**Figure 4:**
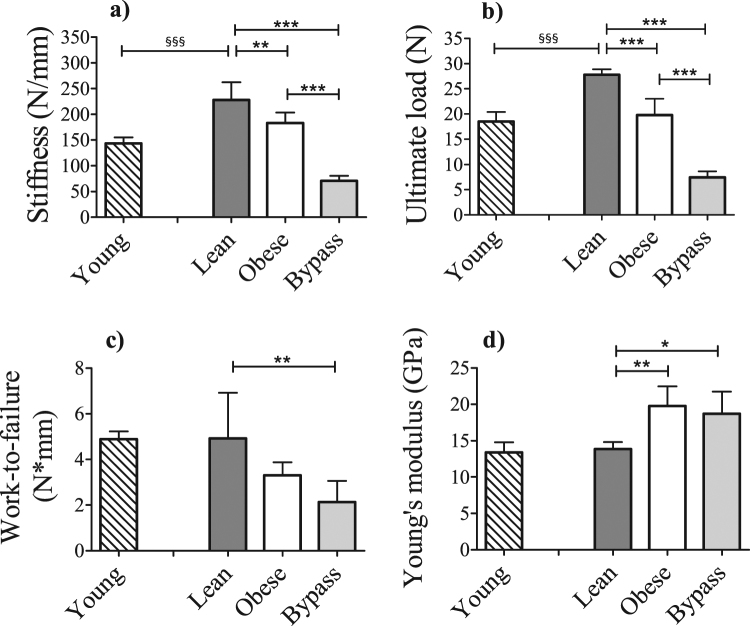
(**a**) Bending stiffness, (**b**) ultimate load, (**c**) work-to-failure, assessed by three-point bending, and (**d**) Young’s modulus, calculated using finite element analysis, of young, lean, obese and bypass mice femora. ^§§§^p < 0.001 when comparing the young and age-matched lean group; *p < 0.05, **p < 0.01, and ***p < 0.001 when comparing the obese, lean and bypass groups. n = 6/group.

In order to determine if the observed alterations in the structural properties in both obese and bypass groups were affecting the biomechanical functionality of bone, we assessed the mechanical properties. Obese mice had less stiff bones than lean controls, but not less than bypass mice, which showed an extremely low stiffness (Fig. 4a). The same results were found for ultimate load (Fig. 4b), revealing that obese and bypass mice possess weaker bones compared to controls. Work-to-failure was lower only in bypass mice compared to lean controls (Fig. 4c). Young mice had significantly less stiff bones and a decreased ultimate load when compared to their ageing counterparts, which was an expected result from the ageing process.

### Changes in Young’s modulus of T2DM and RYGB bone are due to increased accumulation of AGEs

**Figure 5:**
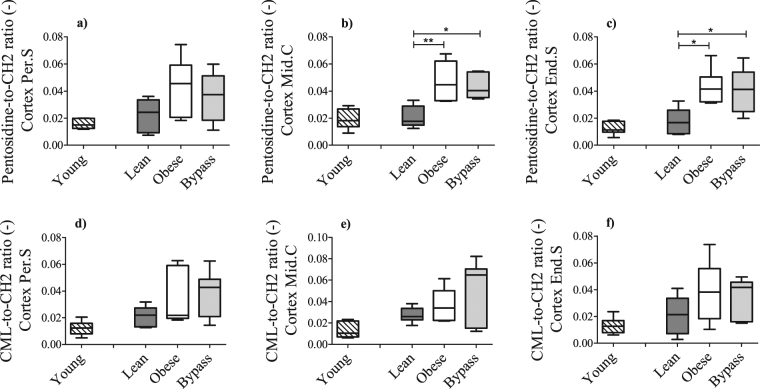
Relative content of the advanced glycation end products (AGEs) pentosidine and CML in periosteal (**a**,**d**), middle (**b**,**e**) and endosteal (**c**,**f**) cortical locations, respectively, for young, lean, obese and bypass groups. The Raman-specific bands for both AGEs were normalized to the CH_2_ band, representative of the organic matrix. Per.S: Periosteal area, Mid.C: Mid-cortical area; End.S: Endosteal area. *p < 0.05 and **p < 0.01 when comparing the obese, lean and bypass groups. n = 6/group.

The material properties of the bones were assessed through the calculation of the Young’s modulus, via finite element modeling (Fig. 4d). This parameter was significantly higher for both obese and bypass groups, compared to controls, indicating altered material properties of the bone tissue. To corroborate this finding, the relative content of the AGEs pentosidine and CML was evaluated using Raman spectroscopy (Fig. 5). Bone tissue from obese and bypass animals showed a clear tendency of increased AGEs accumulation in all cortical areas assessed, and particularly a significant accumulation of pentosidine in the endosteal and mid-cortical areas of both mice groups, compared to lean mice. When the ageing component was evaluated, accumulation of pentosidine and CML species tended to be higher in lean animals compared to the young group, but without reaching statistical significance.

### Compositional characteristics of the endosteal cortex and of trabeculae are altered after RYGB surgery

**Figure 6:**
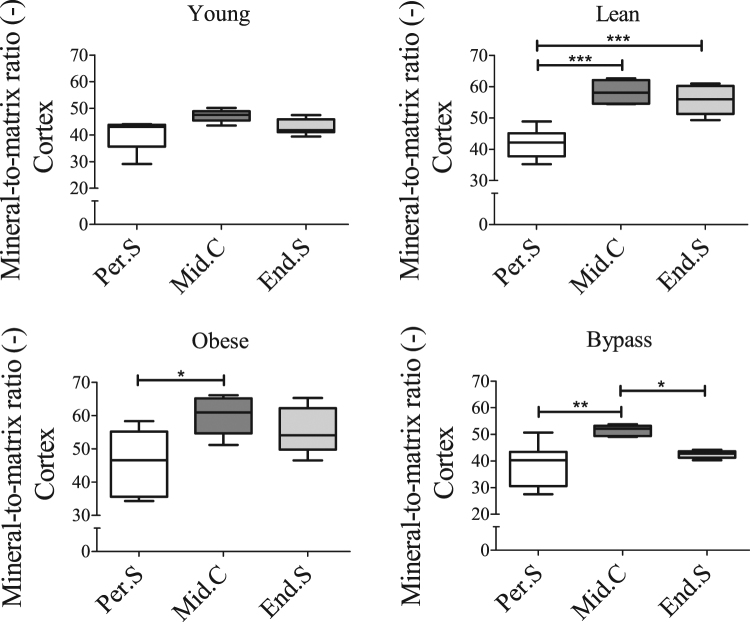
Raman-based mineral-to-matrix ratio at periosteal, middle and endosteal intracortical locations of (**a**) young, (**b**) lean, (**c**) obese and (**d**) bypass femora. 10 point scans were made per cortical location. Per.S: Periosteal area, Mid.C: Mid-cortical area; End.S: Endosteal area. *p < 0.05; **p < 0.01; ***p < 0.001 when comparing the obese, lean and bypass groups. n = 6/group.

**Figure 7:**
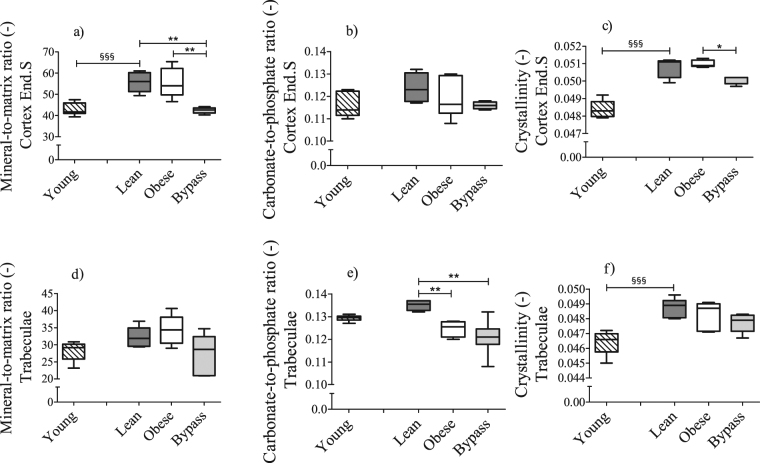
Differences in Raman-based (**a**) mineral-to-matrix ratio, (**b**) carbonate-to-phosphate ratio and (**c**) crystallinity at the endosteal cortical location and (**d**) mineral-to-matrix ratio, (**e**) carbonate-to-phosphate ratio and (**f**) crystallinity of the trabeculae of mice femora between young, lean, obese and bypass mice. End.S: Endosteal area. ^§§§^p < 0.001 when comparing the young and age-matched lean group; *p < 0.05 and **p < 0.01, when comparing the obese, lean and bypass groups. n = 6/group.

In order to investigate if other bone compositional characteristics of the obese and bypass groups were altered, Raman spectroscopy was used again. Obese, bypass and lean groups presented differences in the level of mineralization of the Per.S *vs* Mid.C. Only the bypass group showed significantly lower mineralization of the End.S when compared to the Mid.C (Fig. 6). Mineralization did not differ significantly throughout the cortex of young animals. Moreover, similar results were obtained for the crystallinity values of all groups’ cortical locations (Supplementary Fig. S1), except for the bypass group, which remained uniform among locations. The carbonate-to-phosphate ratio did not vary significantly between any of the locations of the studied groups, except for bypass animals, which showed significantly lower carbonate substitutions in the End.S compared to Mid.C (Supplementary Fig. S1). When comparing Raman-based parameters per cortical location between groups (Fig. 7 and Supplementary Fig. S2), the bypass group presented a significantly lower mineral-to-matrix ratio in the End.S when compared to both lean and obese groups. Relative to the obese group, the crystallinity was lower in the End.S of bypass bones. Mineralization, carbonate substitutions and crystallinity of both lean and obese groups were similar at all cortical locations (Fig. 7 and Supplementary Fig. S2), indicating that T2DM does not impact the bone compositional properties significantly. Mineral-to-matrix and crystallinity were significantly lower in the Mid.C and End.S locations of the young group compared to lean controls.

Regarding the trabecular area (Fig. 7), the carbonate-to-phosphate ratio of bypass and obese groups was significantly lower than in lean controls (p < 0.01). In addition, similar to the cortical area, all physico-chemical parameters of the trabecular bone were increased with ageing, but only crystallinity reached significance.

PLS-DA showed a clear separation of the spectra between young and lean controls for each area assessed (Supplementary Fig. S3). For all bone areas, the carbonate peak was associated with the lean group. Similarly, the young group was mainly associated with the organic phase (Amide I, Amide III and CH2). Furthermore, no clear separation of Raman spectra could be observed between obese *vs* lean, obese *vs* bypass and bypass *vs* lean groups (Supplementary Fig. S4).

### Bone mechanical properties are correlated with macro-architectural parameters - Young’s modulus correlates with pentosidine content

Correlation analyses revealed that both ultimate load and stiffness were correlated with the Ct.Th and Tb.Th in the femora, while work-to-failure correlated positively with trabecular BV/TV, but negatively with Tb.Sp (Table 1). Moreover, the Young’s modulus was found to be positively correlated with the content of pentosidine and almost reached significance with CML accumulation (p = 0.07). These data indicate that both structural and material properties have an impact on bone mechanical performance under T2DM and post-RYGB conditions. Other material and compositional parameters assessed in this study were tested for correlations, but no statistical significance was found in any of the cases.

**Table 1:** Correlations of the mechanical and material parameters with the bone’s architecture and AGEs content assessed in young, lean, obese and bypass femora.

Mechanical/Material	Macro-architectural				AGEs	
	Ct.Th	BV/TV	Tb.Th	Tb.Sp	Pentosidine	CML
Ultimate load	0.987*	NS	0.961*	NS	NS	NS
Work-to-failure	NS	0.991**	NS	−0.950*	NS	NS
Stiffness	0.985*	NS	0.994**	NS	NS	NS
Young’s modulus	NS	NS	NS	NS	0.993**	NS (0.07)

The Pearson correlation coefficient was implemented. Other material and compositional parameters assessed in this study were tested for correlations but no statistical significance was found in any of the cases. For each of the pairings (mechanical/material property parameter vs macro-architectural property/AGEs parameter) significance was indicated as *p<0.05; **p<0.01; NS: No significance.

## Discussion

High-fat diet-induced T2DM generates many severe complications, including skeletal deterioration. Given that RYGB surgery has become the most commonly performed bariatric procedure against morbid obesity and T2DM^25,26^, but has also shown to negatively impact the skeleton, our aim was to determine the possible factors associated with T2DM and subsequent RYGB surgery, leading to deterioration of bone quality and increased fracture risk. To achieve this, structural, mechanical and compositional bone characteristics were assessed in both T2DM and RYGB mouse models.

The assessment of the bone structural characteristics after microCT scanning revealed no osteopenic phenotype in lean controls when compared to the young group. On the contrary, we could see only slightly increased Tb.Th, Tb.S, and in contrast to literature^27,28^, mildly increased trabecular BV/TV. We did observe a physiological age-related pattern of bone growth, significant thickening of the cortex and expansion of the periosteal surface, where bone apposition mainly occurs in normal ageing conditions^28,29^. In the case of obese and bypass mice, the macro-architecture was noticeably altered, evidenced by the thinning of the cortex for both groups. Interestingly, compared to lean controls, the periosteal surface seemed to be diminished in the obese group (lower Ct.OtD), contrary to bypass bones where the endosteal surface was decreased (increased Ct.InD), implying compromised bone expansion in each case. Alterations in bone architecture after RYGB surgery were recently reported, evidencing reduced cortical area in RYGB patients^30^, supporting our results. After *in vivo* ulnar loading applied to C57BL/6-Ins2^Akita^/J (Akita) mice (type 1 diabetes model), another study revealed that periosteal bone formation rate was compromised^31^, in line with the results of our obese mice. The role of osteocytes was particularly highlighted by the authors, as it appears that the mechanosensory response of these cells is compromised under diabetic conditions, thus generating bone turnover alterations in the periosteum^31^.

A previous study from our group demonstrated that obese, T2DM mice showed decreased serum levels of bone turnover biomarkers C-terminal telopeptide of type I collagen (CTX) and osteocalcin (OCN)^32^. Meanwhile, several studies agree that the expression levels of CTX, OCN and/or P1NP in bone are affected after gastric bypass^33,34^. The suggested alteration of the bone remodeling of both mice groups goes in line with the dramatic decrease of trabecular BV/TV found in this study, given the fact that the trabecular compartment is an area more metabolically active than the cortex, and prone to high levels of remodeling^35^.

In this regard, bone turnover markers can be directly altered by variations in the bodyweight of a subject. In the case of our obese mice, a decreased Ct.OtD indicates that the potential influence of the increase in mechanical loading due to the increased weight is not sufficient to trigger mechanisms favoring the formation of new bone tissue^36^. In contrast to the obese group, bypass mice experienced a drastic weight loss, hence a sudden mechanical unloading. This has the capacity to make bone adapt to the decline in mechanical stress by increasing remodeling^37,38^. The lack of an energy-restricted control group is a limitation of our study, since it is difficult to assert that the bone loss observed in our bypass mice is either dependent or independent of weight loss. Nevertheless, there have been several studies in literature reporting that bone loss, evidenced as decreased BMD at the hip (a weight-bearing location), is associated with weight loss after RYGB surgery^30,39^.

Furthermore, it has been previously reported that the presence of AGEs has a negative impact on the osteogenic potential and osteoclastogenesis of progenitor cells. Using human osteoblasts exposed to AGEs *in vitro*, Franke *et al*. (2011) demonstrated low expression of alkaline phosphate and OCN, affecting osteoblastic function and differentiation, and increased RANKL expression, favoring the generation of osteoclastic cells^40^. These data suggest that the accumulation of AGEs found in our obese and bypass mice can contribute to the observed bone loss, through loss of progenitors’ osteogenic potential and enhancement of bone resorption.

Another factor contributing to bone loss through alterations of bone remodeling in our bypass mice can be the nutrients malabsorption caused by the surgery itself. This has been reported before to cause detrimental effects on bone volume and bone cellular homeostasis^34,41^. Elevated expression of PTH (secondary hyperparathyroidism) caused by poor calcium and vitamin D uptake can generate skeletal calcium mobilization to the blood stream in order to stabilize the levels of calcium intestinal absorption, favoring bone resorption^42,43^. All these mechanisms (potentially) triggering bone resorption in our bypass mice suggest that the bone loss process is multifactorial.

Despite this assertion, the lack of a sham-surgery control group represents an extra limitation of our study. Nonetheless, studies in literature have proven that, compared to sham controls, bypass rats showed RYGB-associated bone resorption, with increased expression of CTX, decreased expression of P1NP, and compromised bone structural properties, evidenced as lower trabecular volume, number, thickness, lower cortical volume and thickness^34^.

In this regard, changes of the structural properties, triggered by the impairment of the bone resorption and formation processes, suggest alterations of biomechanical properties and an influence on bone strength^44^. Among the mechanical parameters assessed in this study, the bending stiffness is a parameter closely related to the mineralization of bone tissue^36^. Therefore, it was anticipated to find the femora of young mice less stiff than those of the lean controls^45^. Poorly mineralized bones with low stiffness are expected to have a high ductility^36^. Even though the bypass mice femora showed extreme low stiffness, low work-to-failure values relative to lean mice imply a reduced ultimate displacement and less amount of energy necessary until the bone breaks. Moreover, the fact that ultimate load values are greatly reduced in the bypass group evidences a compromised mechanical performance favoring increased risk of fractures. This is in line with a recent study showing after finite element analysis a decrease in estimated stiffness and ultimate load caused by the disintegration of the cortical and trabecular bone, implying a negative impact on fracture risk in RYGB patients^9^. On the other hand, the impaired periosteal bone apposition found in our study may be reducing the bone strength in the femora of our obese mice, as suggested by the reduced ultimate load, compared to lean controls. This, in addition to the significantly decreased stiffness, and the tendency of decreased work-to-failure, support the findings of different studies evaluating the mechanical performance on mouse models of diabetes^46–48^. Furthermore, correlations between mechanical and structural parameters (Table 1) reflect that the alterations in the macrostructure of both obese and bypass mice femora are strongly influencing the mechanical performance of the bone.

The higher Young’s modulus in the obese and bypass groups compared to lean mice is indicative of an increased material stiffness. As the 3-point bending test takes into account shape and geometry, finite element modeling results suggest that not only the structure but also the material properties are factors responsible for the compromised mechanical performance and diminished bone quality in obese and bypass mice. To elucidate what could be causing the increased Young’s modulus, the presence of AGEs was evaluated by means of Raman microspectroscopy. AGEs accumulation in T2DM bone favors material stiffness and alters bone’s mechanical properties^14,16,49^. Therefore, the elevated levels of AGEs found in our study explain the increase of Young’s modulus in the bones from obese and bypass mice. This is further confirmed by the positive correlation found between the Young’s modulus and the pentosidine content in bone tissue. Furthermore, as a consequence of pentosidine and CML accumulation, fracture toughness initiation and propagation were found decreased in a mice model of type 1 diabetes^50^. These findings support our results in the obese and bypass groups. Even though the RYGB procedure in obese mice was capable of reducing their hyperglycemic state to control levels, the AGEs content was still not normalized, and thus improvement of the bone quality was not achieved. This suggests that either other processes favoring AGEs accumulation (such as oxidative stress) are still strongly present in bypass mice, or that the process of AGEs removal requires an extended period of time, much longer than the 8 weeks post-RYGB applied in the present study^51^.

More parameters related to bone composition at the microscale were analyzed by Raman spectroscopy. Firstly, the Raman parameters varied significantly according to the location within the cortex, also reported by previous studies^23^. These differences were present in all mice groups, but to a much less extent in the young group, implying that notable changes between the locations have not yet manifested. The low mineral and carbonate content of young bones are in agreement with previous studies on rodents, baboons and humans^45,52,53^, evidencing that bone composition changes with ageing. PLS-DA confirmed these results, revealing a clear separation of the 2 groups. The physico-chemical parameters of the cortical locations were highly similar between lean, obese and bypass mice. This was confirmed by the lack of separation of the groups after PLS-DA. Together with the lack of correlation between Raman-based ratios and mechanical parameters, this suggests that, by itself, the composition of bone tissue cannot generate an increased fracture risk in both T2DM subjects and RYGB-intervened subjects, but should be seen as a contributor along with other possible detrimental factors. An exception was seen in the endosteal surface of the bypass mice femora, where significantly lower mineral-to-matrix ratio and crystallinity were observed compared to the bones from lean and obese animals. It is noteworthy that mainly all compositional alterations found in the cortex of the bypass group femora correspond to the endosteal location, which was found to be severely compromised according to the microstructural analysis.

The high levels of material stiffness (Young’s modulus) despite the lower level of mineralization of the femora from the bypass group, further suggests the influence of the AGEs in the stiffening of the tissue. In addition, no correlation could be established between the Raman-based ratios and material stiffness in our study. Thus, although the endosteal cortical area of the bypass group seemed to be poorly mineralized, other mechanisms different from bone mineralization (like the AGEs accumulation and macro-architectural alterations) must explain the increased fracture risk in subjects after RYGB surgery. Likewise, the degree of mineralization measured by Raman spectroscopy cannot explain the reduced stiffness calculated by 3-point bending in the obese group, since we found no differences of mineral-to-matrix ratio in cortical and trabecular areas of obese mice compared to lean controls. This differs with a previous study on Zucker diabetic Sprague-Dawley rats by Hammond *et al*. (2013), which showed increased values of mineral-to-matrix ratio relative to controls^54^. However, the authors also found a decreased ash fraction in obese rats’ tibiae, which led them to discuss the possibility that the T2DM-induced accumulation of AGEs was impeding the collagen structure to freely vibrate, reducing the signal of the collagen bands and potentially overestimating mineral-to-matrix values. We cannot discard the possibility that this may be happening also in our obese and bypass mouse models.

Differences in the rest of the Raman-based parameters were not found between lean and obese mice, implying that bone composition between these 2 groups is alike. These data support the findings made by Hammond *et al*., showing no significant differences between control and T2DM rats for carbonate-to-phosphate ratio and crystallinity^54^. The only Raman-based parameter that was found to be significantly different between obese and lean bone samples in the present study was the carbonate-to-phosphate ratio, lower in the trabecular area of the femora from obese mice. As mentioned before, this same area showed trabecular bone loss compared to lean animals after microCT scanning. Thus, as previously discussed in this section, it is possible that alterations in the process of bone remodeling taking place in the femora of our obese mice are causing changes in the composition of the apatite lattice in terms of substitution of carbonate ions.

In conclusion, the observed changes at the cortical periosteal (obese) and endosteal (bypass) surfaces, in addition to alterations of the trabecular area, imply that bone turnover abnormalities are taking place in T2DM- and post-RYGB bones. These alterations in the bone structural properties are negatively affecting the biomechanical functionality of bones from obese and bypass mice, and thus are increasing the risk of bone fracture in these mice groups. Furthermore, bone fracture mechanics are influenced by compromised material properties in both obese and bypass groups due to the increased accumulation of AGEs, of which pentosidine was associated with an increased Young’s modulus compared to controls. Overall, the etiology of the increased fracture risk in both T2DM- and RYGB bones appears to be mainly driven by material- and macro-structural-influenced biomechanical dysfunctionality.

## Materials and Methods

### Animals

Animal experimental procedures were performed in accordance with the Belgian legislation, the ARRIVE guidelines and the ILAR Guide to the Care and Use of Experimental Animals, and they were approved by the Ethics Committee of the University of Leuven (P101/2014). Six-weeks-old male mice, substrain C57BL/6, were obtained from Janvier Labs (Le Genest-Saint-Isle, France). Four to 5 mice per cage were housed and allowed to acclimatize for 2 weeks before the start of the study. Food and water were available *ad libitum*. A 12:12 h light-dark cycle was implemented and room temperature was kept at 22 °C.

Mice were randomly divided into 2 groups, namely a low-fat diet (10 kcal% fat - aged-matched lean group (**lean**), n = 6) and high-fat diet (60 kcal% fat - diet-induced obese group (**obese**), n = 12) group. The diets (Research Diets, Inc. – New Brunswick, NJ, USA – Low-fat diet: D12450-B; High-fat diet: D12492; open source) were administered to lean and obese mice for a total period of 22 weeks (age of sacrifice: 30 weeks old), allowing the DIO animals to reach a diabetic condition. In order to corroborate that potential changes in the bone properties of the experimental mice groups are not occurring (solely) due to ageing effects, an additional group of young healthy 8-weeks-old mice were added as baseline control (group **young**, n = 6). At the end of the experiment, body weight and glucose levels were determined.

Six obese mice were randomly assigned to a new group after 14 weeks of high-fat diet intake, and were subject to RYGB surgery (group **bypass**). The RYGB surgical procedure was performed under carprofen analgesia (3 µg/g bodyweight) and 2–3% isoflurane anesthesia. Briefly, a 2–2.5 cm midline abdominal incision was made and the esophagus, stomach and proximal jejunum were dissected using cotton tip applicators and fine scissors or forceps. The jejunum was transected 3 cm below the ligament of Treitz and an end-to-side jejunojejunostomy was created with a 9–0 nylon separated suture. Next, the stomach was transected, the jejunum brought up to the stomach to perform an end-to-side gastrojejunostomy with 9–0 nylon separated suture, creating an alimentary limb. At the end, the midline incision was closed and the mice were recovered on a water-circulated heating pad. The mice had *ad libitum* access to water and normal chow diet from postoperative day 1 to 6, where after they were reinstated on high-fat diet for 7 additional weeks (age of sacrifice: 30 weeks old), to allow for their recuperation and ensure that enough time has passed for the potential effects of the RYGB surgery to take place.

### Bone tissue collection

Animals were euthanized by means of carbon dioxide administration. Since fixation or embedding of bone samples can alter the compositional properties of the tissue, interfering with the Raman bone-related spectral parameters^55^ or affecting the mechanical properties, we decided to preserve the samples by snap-freezing^56^. Femora were snap-frozen through liquid nitrogen immersion, wrapped in aluminum foil and stored at −80 °C.

### MicroCT acquisition and image analysis

Left femora (n = 6 per group) were scanned at 8 µm voxel size to image the full sample within 1 scan, using the Phoenix NanoTom S (GE Measurement and Control Solutions, Germany). The source was set at 60 kV and 210 µA, and an aluminum filter of 0.2 mm was applied. Application of the ‘fast mode’ settings (i.e. exposure time 500 ms, frame averaging 1 and skip 0) and acquisition of 2400 radiographs resulted in a scanning time of 20 minutes per sample.

Images were analyzed using CTAn software (Bruker MicroCT, Kontich, Belgium). Two separate areas of the bone (cortical and trabecular area) were analyzed. Both areas covered 1.2 mm of bone length (150 cross-sections). For the trabecular area, the area started 800 µm below the growth plate and continued downwards. The trabecular structure was manually included inside a region of interest (ROI). Firstly, images within the ROI underwent a binarization step (Otsu^57^), followed by noise removal by a closing operation (round kernel, radius 1, 3D space) and a double despeckling step (removal of black and white speckles respectively, 3D space, less than 25 voxels), as described previously^32^. 3D analysis of the processed datasets allowed determining the following parameters: trabecular volume fraction (BV/TV, %), trabecular thickness (Tb.Th, µm) and trabecular separation (Tb.Sp, µm). For the cortical area, the area started at the fibula-to-tibia insertion point and continued upwards. The ROI fitting the cortical bone was drawn automatically using an in-house developed protocol. After binarization of the cross-sections using Otsu segmentation, noise and cortical porosity were removed using despeckling as previously described^32^. In order to determine the cortical thickness (Ct.Th, µm), binarized images went through a process of “skeletonization” based on “distance transform” algorithms^58^ to identify the medial axis of each cross-section, on the model-independent technique from Hildebrand and Ruegsegger^59^. Furthermore, a “sphere-fitting” process^59^ was carried out for all voxels comprising the established axis. Finally, an average value of all fitted spheres was obtained. The same procedure was used to determine the cortical outer (Ct.OtD, µm) and cortical inner diameter (Ct.InD, µm), analyzing the binarized images with a filled marrow cavity and images of the marrow cavity space, respectively.

### Three-point bending test

Bone stiffness, ultimate load and work-to-failure were calculated from load *versus* displacement curves using 3-point bending tests on left femora (n = 6 per group), following a protocol described before^60^. Briefly, testing was performed using the BOSE Test Bench (LM1, EnduraTEC Systems Group, Bose Corp., Minnetonka, MN, USA). The span length was 6 mm whereas the radius of curvature of each support was 2 mm. The bones were placed with their anterior surface on the two supports and were subjected to a 1 N stabilizing preload and 2 conditioning cycles prior to loading until failure at a rate of 0.1 mm/s.

### Finite element analysis

Finite element analyses, simulating the 3-point bending tests, were performed based on the microCT data using a validated approach^61^. Briefly, an automated alignment routine turned the reconstructed femora such that their anterior surface was in contact with two supports as in the experimental setup described above. Finite element models were created from the aligned femora using a standard voxel conversion technique, hence, each voxel from the microCT reconstruction was converted to a hexahedral element in the finite element model. Boundary conditions mimicking the 3-point bending test were applied^61^. All elements in the finite element models were given an identical, arbitrary tissue modulus of 10 GPa and a Poisson ratio of 0.3. Assuming linear-elastic material behavior, the models were solved using ParOSol^62^, a dedicated micro-finite element solver running on a supercomputer (CSCS, Lugano, Switzerland). The finite element sample stiffness was calculated as the finite element calculated force divided by the prescribed displacement. The bone tissue Young’s modulus was determined by linear scaling of the finite element sample stiffness to the experimentally measured stiffness^63^.

### Raman spectroscopy

Right femora (n = 6 for each group) were thawed at room temperature and cut in half along the longitudinal axis using a diamond saw. Femora were analyzed using Raman microspectrometer SENTERRA (Bruker) equipped with a laser of λ = 720 nm. Raman spectra were acquired with a 100× objective and a total integration time of 135 sec (45 secs (×3)). The spectral range was 400–1780 cm^−1^ with a spectral resolution of 3–5 cm^−1^. Baseline correction and peak intensities were done using the OPUS software 7.2 (Bruker).

Mineral-to-matrix ratio determines the mineralization degree of the sample. It was calculated from the ratio of the phosphate symmetric stretch band (960 cm^−1^) to the CH_2_ band (1450 cm^−1^). Crystallinity, a measure of the size and perfection of the apatite lattice, was calculated as the inverse of the full width at half maximum (FWHM) of the phosphate symmetric stretch band, using the OriginPro 2017 software (OriginLab, Massachusetts, USA). The carbonate-to-phosphate ratio represents the carbonate substitutions for phosphate positions in bone. It was calculated as the ratio of the type-B carbonate band (1070 cm^−1^) and the phosphate symmetric stretch band. The intensities of the Raman-specific bands previously reported for pentosidine (~1495 cm^−1^) and carboxymethyl-lysine (CML; ~1150 cm^−1^) were obtained and normalized to the CH_2_ band to evaluate AGEs accumulation^50^.

The physico-chemical variables are dependent on the location in the cortex^23^. Thus, the cortical bone was analyzed at 3 specific ROIs (Supplementary Fig. S5): (i) periosteal surface (Per.S), (ii) mid-cortical area (Mid.C) and (iii) endosteal surface (End.S). Acquisitions were made at the mid-diaphyseal region. Regarding the trabecular bone, acquisitions were performed at the distal epiphysis of the femora. Ten spectra were acquired per cortical ROI and 20 spectra in the trabecular area. Each physico-chemical parameter was averaged over the 10–20 spectra per ROI for each sample.

Partial least squares discriminant analysis (PLS-DA) was carried out to find the specific Raman spectral features and to sharpen the separation between the studied groups. The analysis was done using spectral datasets of known class membership composed of 2 known classes (young *vs* lean, obese *vs* lean, obese *vs* bypass and lean *vs* bypass) per ROI (Per.S, Mid.C, End.S, Trabecular), using a methodology previously described^64^. PLS-DA was performed using the PLS Toolbox v6.7 (Eigenvector Research, Inc., Wenatchee, WA, USA) in Matlab (Mathworks Inc., Natick, MA, USA).

### Statistical analysis

Data for all parameters are expressed as group means ± standard deviation. Analysis of normality of the data was performed using the D’Agostino-Pearson test and the presence of outliers was tested using the Dixon test. Equality of variances was assumed when the F-test (normally distributed) or Levene’s test (not normally distributed) revealed values of p > 0.05. The young group was independently compared to the lean group using a two-tailed unpaired t-test (normally distributed) or a Kruskal-Wallis test (not normally distributed) with equal or unequal variance depending on the F-test or Levene’s test respectively. Since the obese and bypass mouse models need a combination of time (ageing) and diet to generate the skeletal impairments, we cannot assume that these factors are truly independent. For this reason, we could not compare these groups with the young mice group. For comparison of the obese, lean and bypass groups, a one-way ANOVA test was used. All statistical analysis was performed using GraphPad PRISM (GraphPad software, La Joya, CA, USA). Significance is indicated in the graphs as follows: ^§^p < 0.05, ^§§^p < 0.01, and ^§§§^p < 0.001 when comparing the young and lean group; *p < 0.05, **p < 0.01, and ***p < 0.001 when comparing the obese, lean and bypass groups. In order to evaluate correlations between the assessed parameters, a Pearson correlation coefficient test was used for data displaying normal distribution with p < 0.05 for significant correlations.

## Electronic supplementary material

Supplementary data

## References

[1] Wang C-Y, Liao JK (2012). A mouse model of diet-induced obesity and insulin resistance. Methods Mol. Biol..

[2] Andrikopoulos S, Blair AR, Deluca N, Fam BC, Proietto J (2008). Evaluating the glucose tolerance test in mice. Am. J. Physiol. Endocrinol. Metab..

[3] Farr JN (2014). *In vivo* assessment of bone quality in postmenopausal women with type 2 diabetes. J. Bone Miner. Res..

[4] Retzepi M, Donos N (2010). The effect of diabetes mellitus on osseous healing. Clin. Oral Implants Res..

[5] Pizzorno L (2016). Bariatric Surgery: Bad to theBone, Part 1. Integr. Med. (Encinitas)..

[6] Courcoulas AP (2015). Three-Year Outcomes of Bariatric Surgery vs Lifestyle Intervention for Type 2 Diabetes Mellitus Treatment. JAMA Surg..

[7] Esposito K, Maiorino MI, Petrizzo M, Bellastella G, Giugliano D (2014). The effects of a Mediterranean diet on the need for diabetes drugs and remission of newly diagnosed type 2 diabetes: Follow-up of a randomized trial. Diabetes Care.

[8] Raoof, M., Näslund, I., Rask, E. & Szabo, E. Effect of Gastric Bypass on Bone Mineral Density, Parathyroid Hormone and Vitamin D: 5 Years Follow-up. *Obes*. *Surg*. 1–5, 10.1007/s11695-016-2114-3 (2016). 10.1007/s11695-016-2114-3 26926187

[9] Frederiksen KD (2016). Bone Structural Changes and Estimated Strength After Gastric Bypass Surgery Evaluated by HR-pQCT. Calcif. Tissue Int..

[10] Cheng N-C, Hsieh T-Y, Lai H-S, Young T-H (2016). High glucose-induced reactive oxygen species generation promotes stemness in human adipose-derived stem cells. Cytotherapy.

[11] Schwartz AV (2011). Association of BMD and FRAX score with risk of fracture in older adults with type 2 diabetes. JAMA.

[12] Yamamoto M, Yamaguchi T, Yamauchi M, Kaji H, Sugimoto T (2009). Diabetic patients have an increased risk of vertebral fractures independent of BMD or diabetic complications. J. Bone Miner. Res..

[13] Masahiro Yamamoto. Insights into bone fragility in diabetes: The crucial role of bone quality on skeletal strength. *Endocr*. *J*. **62**, 299–308 (2015). 10.1507/endocrj.EJ15-0129 25797364

[14] Saito M, Fujii K, Mori Y, Marumo K (2006). Role of collagen enzymatic and glycation induced cross-links as a determinant of bone quality in spontaneously diabetic WBN/Kob rats. Osteoporos. Int..

[15] Wang X, Shen X, Li X, Mauli Agrawal C (2002). Age-related changes in the collagen network and toughness of bone. Bone.

[16] Tanaka KI, Yamaguchi T, Kanazawa I, Sugimoto T (2015). Effects of high glucose and advanced glycation end products on the expressions of sclerostin and RANKL as well as apoptosis in osteocyte-like MLO-Y4-A2 cells. Biochem. Biophys. Res. Commun..

[17] Saito M, Kida Y, Kato S, Marumo K (2014). Diabetes, collagen, and bone quality. Curr. Osteoporos. Rep..

[18] Shu L (2015). High-Fat Diet Causes Bone Loss in Young Mice by Promoting Osteoclastogenesis Through Alteration of the Bone Marrow Environment. Calcif. Tissue Int..

[19] Inzana JA (2013). Immature mice are more susceptible to the detrimental effects of high fat diet on cancellous bone in the distal femur. Bone.

[20] Donnelly E, Chen DX, Boskey AL, Baker SP, Van Der Meulen MCH (2010). Contribution of mineral to bone structural behavior and tissue mechanical properties. Calcif. Tissue Int..

[21] Guo X, Kim C (2002). Mechanical consequence of trabecular bone loss and its treatment: a three-dimensional model simulation. Bone.

[22] Mandair GS, Morris MD (2015). Contributions of Raman spectroscopy to the understanding of bone strength. Bonekey Rep..

[23] Donnelly E, Boskey AL, Baker SP, van der Meulen MCH (2010). Effects of tissue age on bone tissue material composition and nanomechanical properties in the rat cortex. J. Biomed. Mater. Res. A.

[24] Burket J (2011). Microstructure and nanomechanical properties in osteons relate to tissue and animal age. J. Biomech..

[25] Schauer PR (2014). Bariatric Surgery versus Intensive Medical Therapy for Diabetes — 3-Year Outcomes. N. Engl. J. Med..

[26] Vasas P (2016). Mid-Term Remission of Type 2 Diabetes Mellitus After Laparoscopic Roux En-Y Gastric Bypass. World J. Surg..

[27] Glatt V, Canalis E, Stadmeyer L, Bouxsein ML (2007). Age-Related Changes in Trabecular Architecture Differ in Female and Male C57BL/6J Mice. J. Bone Miner. Res..

[28] Buie HR, Moore CP, Boyd SK (2008). Postpubertal architectural developmental patterns differ between the L3 vertebra and proximal tibia in three inbred strains of mice. J. Bone Miner. Res..

[29] Halloran BP (2002). Changes in bone structure and mass with advancing age in the male C57BL/6J mouse. J. Bone Miner. Res..

[30] Stein EM (2013). Bariatric surgery results in cortical bone loss. J. Clin. Endocrinol. Metab..

[31] Parajuli A (2015). Bone’s responses to mechanical loading are impaired in type 1 diabetes. Bone.

[32] Kerckhofs G (2016). Changes in bone macro- and microstructure in diabetic obese mice revealed by high resolution microfocus X-ray computed tomography. Sci. Rep..

[33] Yu EW (2016). Cortical and trabecular deterioration in mouse models of Roux-en-Y gastric bypass. Bone.

[34] Canales BK, Schafer AL, Shoback DM, Carpenter TO (2014). Gastric bypass in obese rats causes bone loss, vitamin D deficiency, metabolic acidosis, and elevated peptide YY. Surg. Obes. Relat. Dis..

[35] Clarke B (2008). Normal Bone Anatomy and Physiology. Clin. J. Am. Soc. Nephrol..

[36] Turner CH (2006). Bone strength: Current concepts. Ann. N. Y. Acad. Sci..

[37] Frost HM (1987). Bone "mass" and the "mechanostat": a proposal. Anat. Rec..

[38] Folli F (2012). Bariatric surgery and bone disease: from clinical perspective to molecular insights. Int. J. Obes. (Lond)..

[39] Fleischer J (2008). The Decline in Hip Bone Density after Gastric Bypass Surgery Is Associated with Extent of Weight Loss. The Journal of clinical endocrinology and metabolism.

[40] Franke S (2011). Advanced glycation end products affect growth and function of osteoblasts. Clin. Exp. Rheumatol..

[41] Xanthakos SA (2009). Nutritional Deficiencies in Obesity and After Bariatric Surgery. Pediatric Clinics of North America.

[42] Johnson JM (2005). Effects of gastric bypass procedures on bone mineral density, calcium, parathyroid hormone, and vitamin D. J. Gastrointest. Surg..

[43] Valderas JP (2009). Increase of bone resorption and the parathyroid hormone in postmenopausal women in the long-term after roux-en-y gastric bypass. Obes. Surg..

[44] Pérez-Castrillón JL (2012). The deleterious effect of bariatric surgery on cortical and trabecular bone density in the femurs of non-obese, type 2 diabetic Goto-Kakizaki rats. Obes. Surg..

[45] Brodt MD, Ellis CB, Silva MJ (1999). Growing C57Bl/6 mice increase whole bone mechanical properties by increasing geometric and material properties. J. Bone Miner. Res..

[46] Hamann C (2014). Effects of parathyroid hormone on bone mass, bone strength, and bone regeneration in male rats with type 2 diabetes mellitus. Endocrinology.

[47] Ionova-Martin SS (2011). Changes in cortical bone response to high-fat diet from adolescence to adulthood in mice. Osteoporos. Int..

[48] Maycas M (2016). PTHrP-Derived Peptides Restore Bone Mass and Strength in Diabetic Mice: Additive Effect of Mechanical Loading. J. Bone Miner. Res..

[49] Karim L, Bouxsein ML (2016). Effect of type 2 diabetes-related non-enzymatic glycation on bone biomechanical properties. Bone.

[50] Rubin, M. R. *et al*. Advanced glycation endproducts and bone material properties in type 1 diabetic mice. *PLoS One* **11** (2016). 10.1371/journal.pone.0154700 PMC4854398 27140650

[51] Gkogkolou P, Bohm M, Böhm M (2012). Advanced glycation end products: key players in skin ageing?. Gkogkolou, Paraskevi Bohm, Markus.

[52] Freeman JJ, Wopenka B, Silva MJ, Pasteris JD (2001). Raman spectroscopic detection of changes in bioapatite in mouse femora as a function of age and *in vitro* fluoride treatment. Calcif. Tissue Int..

[53] Akkus O, Adar F, Schaffler MB (2004). Age-related changes in physicochemical properties of mineral crystals are related to impaired mechanical function of cortical bone. Bone.

[54] Hammond MA, Gallant MA, Burr DB, Wallace JM (2013). Nanoscale changes in collagen are reflected in physical and mechanical properties of bone at the microscale in diabetic rats. Bone.

[55] Yeni YN, Yerramshetty J, Akkus O, Pechey C, Les CM (2006). Effect of fixation and embedding on Raman spectroscopic analysis of bone tissue. Calcif. Tissue Int..

[56] McElderry J-DP, Kole MR, Morris MD (2011). Repeated freeze-thawing of bone tissue affects Raman bone quality measurements. J. Biomed. Opt..

[57] Otsu N (1979). A threshold selection method from gray-level histograms. IEEE Trans. Syst. Man. Cybern..

[58] Remy E, Thiel E (2002). Medial axis for chamfer distances: Computing look-up tables and neighbourhoods in 2D or 3D. Pattern Recognit. Lett..

[59] Hildebrand T, Rüegsegger P (1997). A new method for the model-independent assessment of thickness in three-dimensional images. J. Microsc..

[60] Callewaert F (2010). Sexual dimorphism in cortical bone size and strength but not density is determined by independent and time-specific actions of sex steroids and IGF-1: evidence from pubertal mouse models. J. Bone Miner. Res..

[61] van Lenthe GH, Voide R, Boyd SK, Müller R (2008). Tissue modulus calculated from beam theory is biased by bone size and geometry: Implications for the use of three-point bending tests to determine bone tissue modulus. Bone.

[62] Flaig C, Arbenz P (2011). A scalable memory efficient multigrid solver for micro-finite element analyses based on CT images. in. Parallel Computing.

[63] van Rietbergen B, Weinans H, Huiskes R, Odgaard A (1995). A new method to determine trabecular bone elastic properties and loading using micromechanical finite-element models. J. Biomech..

[64] Pascart T (2016). Bone Samples Extracted from Embalmed Subjects Are Not Appropriate for the Assessment of Bone Quality at the Molecular Level Using Raman Spectroscopy. Anal. Chem..

