# Supplementary material for: Unraveling the compromised biomechanical performance of type 2 diabetes- and Roux-en-Y gastric bypass bone by linking mechanical-structural and physico-chemical properties
Source: Sci Rep. 2018 Apr 12;8:5881. doi: 10.1038/s41598-018-24229-x (PMC5897570; doi:10.1038/s41598-018-24229-x)

Unraveling the compromised biomechanical performance of type 2 diabetes- and Roux-en-Y gastric bypass bone by linking mechanical-structural and physico-chemical properties

Carlos Marin [(1,2,3)], Georgios Papantonakis [(4)], Kathleen Sels [(3)], G. Harry van Lenthe [(4)], Guillaume Falgayrac [(5)], Roman Vangoitsenhoven [(6)], Bart Van der Schueren [(6)], Guillaume Penel [(5)], Frank Luyten [(1,2)], Katleen Vandamme[a] [(2,3)], Greet Kerckhofs[a*(1,2,7)]

[a]Shared last authorship

(1) Skeletal Biology and Engineering Research Center, Department of Development and Regeneration, KU Leuven, Leuven, Belgium.

(2) Prometheus - Division of Skeletal Tissue Engineering Leuven, KU Leuven, Leuven, Belgium.

(3) Biomaterials – BIOMAT, Department of Oral Health Sciences, KU Leuven, Leuven, Belgium.

(4) Biomechanics Section, Department of Mechanical Engineering, KU Leuven, Leuven, Belgium.

(5) Lille University, Littoral Côte d'Opale University, EA 4490, PMOI, Physiopathologie des Maladies Osseuses Inflammatoires, F-59000 Lille, France.

(6) Clinical and Experimental Endocrinology, Department of Clinical and Experimental Medicine, KU Leuven, Leuven, Belgium.

(7) Biomechanics lab, Institute of Mechanics, Materials, and Civil Engineering, UCLouvain, Belgium.

* Corresponding author: Prof. Greet Kerckhofs; Biomechanics Lab - Institute of Mechanics, Materials, and Civil Engineering, UCLouvain, Belgium; Tel: (+32) 10 472275; Email: greet.kerckhofs@uclouvain.be

SUPPLEMENTARY MATERIAL

Supplementary Fig. S1. Raman-based carbonate-to-phosphate ratio and crystallinity of young, lean, obese and bypass groups, at periosteal, middle and endosteal intracortical locations of the femora. 10 point scans were made per cortical location. Per.S: Periosteal area, Mid.C: Mid-cortical area; End.S: Endosteal area. *p<0,05; **p<0,01. n = 6/group

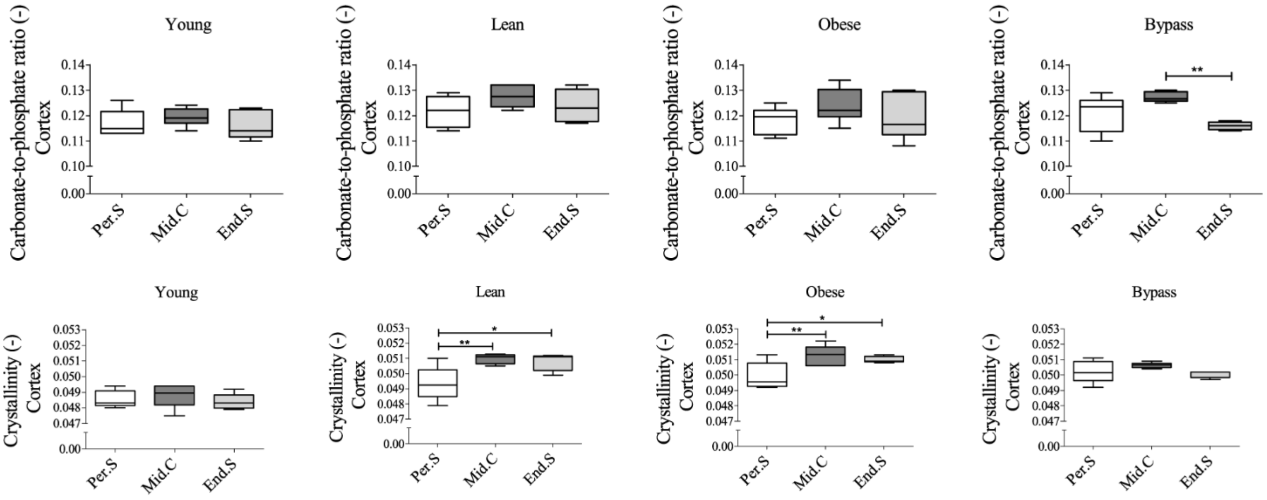

Supplementary Fig. S2. Differences in Raman-based a) mineral-to-matrix ratio, b) carbonate-to-phosphate ratio and c) crystallinity at the periosteal cortical location, and Raman-based d) mineral-to-matrix ratio, e) carbonate-to-phosphate ratio and f) crystallinity at the mid-cortex, compared between the different mice groups. Per.S: Periosteal area, Mid.C: Middle cortical area. § $p<0.05$, §§ $p<0.01$, and §§§ $p<0.001$ when comparing the young and lean group; *$p<0.05$ when comparing the obese, lean and bypass groups *$p<0,05$; **$p<0,01$; ***$p<0,001$. n = 6/group

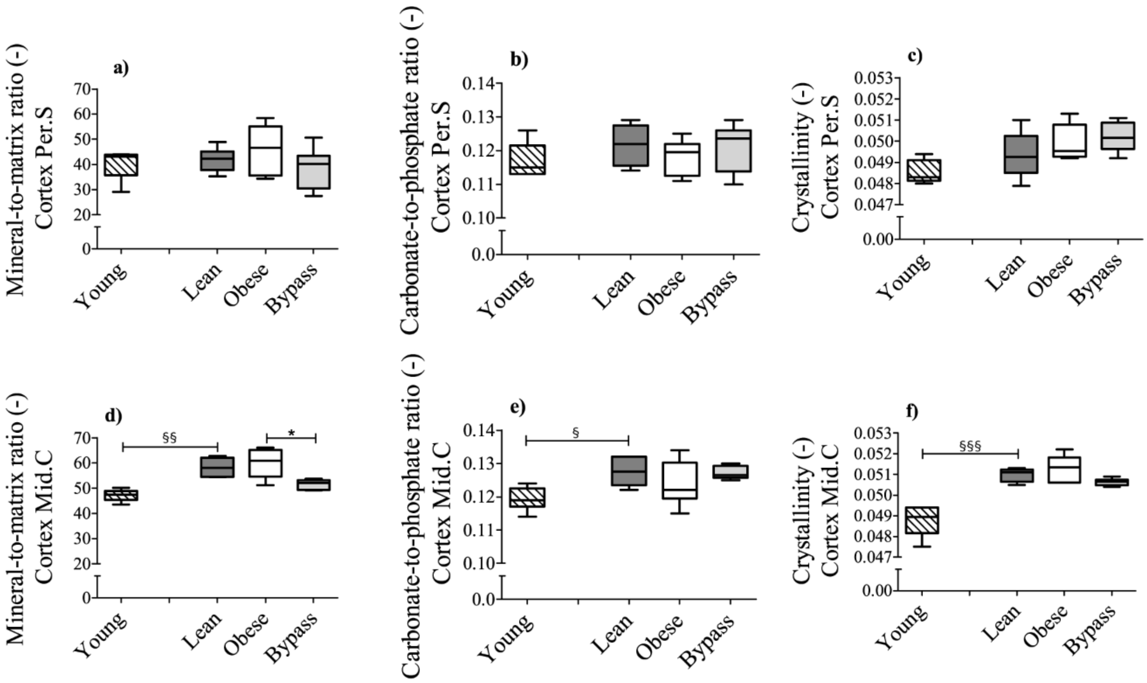

Supplementary Fig. S3. Partial least squares discriminant analysis (PLS-DA) of Raman spectra from young (blue triangles) and lean (red diamonds): a) cortical End.S area and b) Trabecular area. Upper panels represent score plots generated with approx. 180 (cortical) and 120 (trabecular) spectra from each group, showing their predicted distribution (lean: positive, young: negative). Lower panels represent regression vectors depicting the intensity of the correlation between the Raman bands in the studied spectra, and the mice groups compared (lean: positive, young: negative). The dashed line represents the discrimination border between the 2 groups. n = 6/group

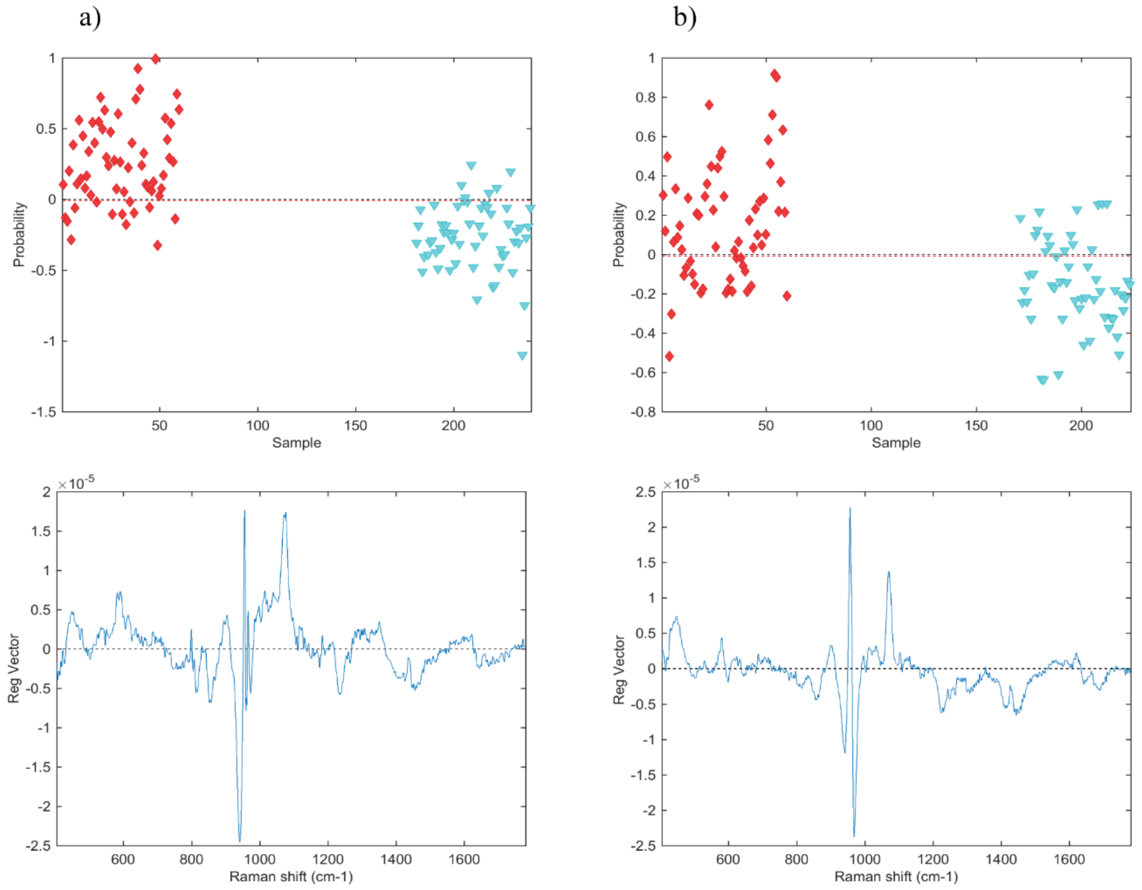

Supplementary Fig. S4. Partial least squares discriminant analysis (PLS-DA) of Raman spectra from a) lean vs obese, b) bypass vs obese and c) lean vs bypass in the cortical End.S of mice femora. The panels represent score plots generated with (approx.) 180 spectra from each group, showing their predicted distribution (lean: always positive; obese: always negative; bypass: positive vs obese, negative vs lean). The dashed line represents the discrimination border between the 2 groups compared. Red diamonds: lean samples; Blue triangles: obese samples; Green squares: bypass samples. n = 6/group.

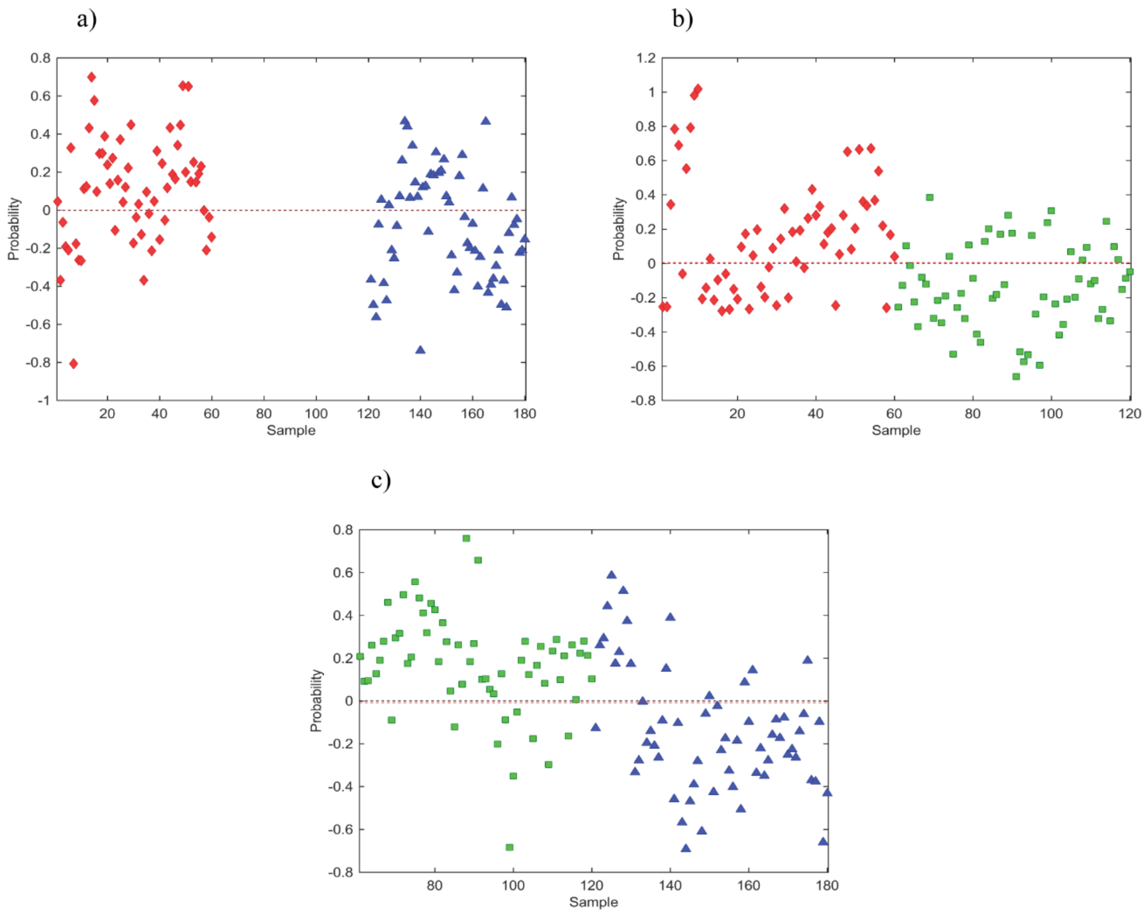

Supplementary Fig. S5. Single point scan locations (red circles) in a) cortical area and b) trabecular area of mice femora, assigned for Raman spectroscopy. Cortex scanning was divided into 3 different intracortical locations. Per.S: Periosteal surface, Mid.C: Mid-cortical area; End.S: Endosteal surface; Trab: trabecular area. Scale bars: 50 μm.

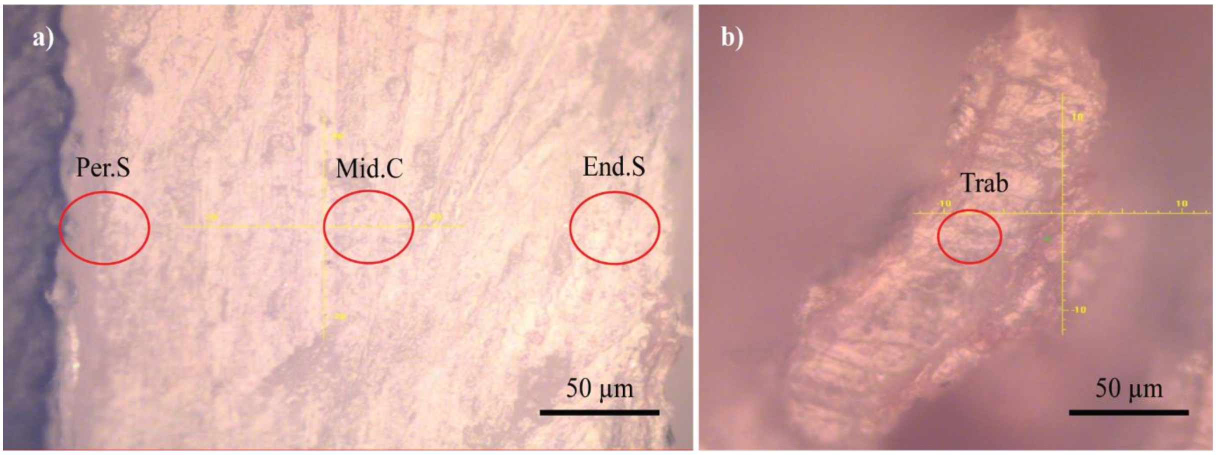

Supplement: Supplementary file 1 — Supplementary data [file 41598_2018_24229_MOESM1_ESM.pdf]
